# Posterior fossa ruptured dermoid cyst presenting with hydrocephalus

**DOI:** 10.17712/nsj.2016.4.20160280

**Published:** 2016-10

**Authors:** Abrar A. Wani, Uday S. Raswan, Nayil K. Malik, Altaf U. Ramzan

**Affiliations:** *From the Departments of Neurosurgery, Sher-i-Kashmir Institute of Medical Sciences, Soura, Srinagar, India*

## Abstract

Dermoid cysts are rare, benign lesions of embryological origin that represent 0.1-0.7% of all intracranial tumors. They are mainly located in the supra tentorial space, especially in the parasellar region. Their location in the posterior fossa remains uncommon. Rupture of intracranial dermoid cysts is a rare phenomenon. We present a case of dermoid cyst, which had ruptured into ventricular system. Computed Tomography and MRI revealed fat in the fourth ventricle, prepontine cistern, and cerebellomedullary cistern. Hydrocephalus was noted. We performed right ventriculo-peritoneal shunt on which patient improved and he continues to remain asymptomatic one year after.

Dermoid cysts are rare, benign lesions of embryological origin that represent 0.1-0.7% of all intracranial tumors. They are mainly located in the supra tentorial space, especially in the parasellar region. Their location in the posterior fossa remains uncommon.^1^ Ruptured posterior fossa dermoid cyst presenting with hydrocephalus has not been reported so far in the literature despite varied presentations of dermoid cysts.^1^-^3^ This is the first reported case of dermoid cyst with such a varied presentation. Our objective is to emphasize the readers on one of the rare causes of hydrocephalus being tumor rupture into ventricular system and treatment being just ventricular drainage in case main tumor has completely disappeared after rupture.

## Case Report

**Figure 1:**
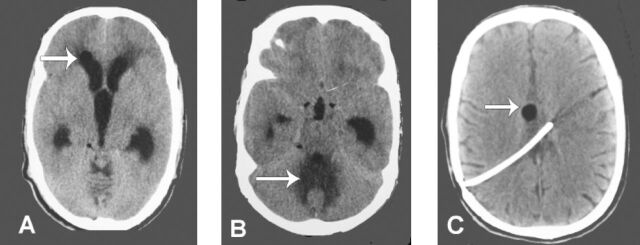
Pre-op non-contrast CT brain **a)** Features of hydrocephalus with marker showing fat in lateral ventricle, **b)** Changes in the intensity in vermis, possible site of dermoid cyst after rupture and **C)** Post-op CT scan decompressed ventricle post–operatively with shunt in situ and marker showing fat.

**Figure 2:**
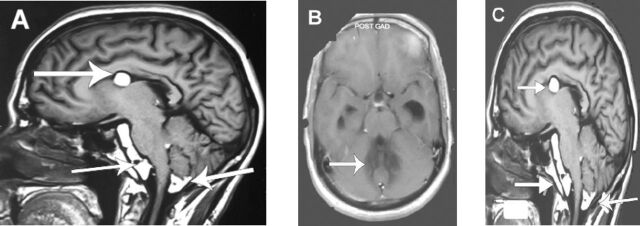
Pre-op MRI with contrast **a)** Pre–op (T1 MRI)- Fat in the prepontine, cerebellomedullary cistern and fourth ventricle with hydrocephalus, **b)** Marked area shows intensity changes in vermis (non enhancement on contrast) possible site of dermoid cyst rupture **c)** post-op MRI (T1) fat in the same regions with ventricle decompression. Also fat visualised in the lateral ventricle.

A 30-year-old male was admitted to the Neurosurgical Emergency Unit complaining of severe headache, vomiting, and diplopia. Neurological examination revealed a conscious patient with gait ataxia and horizontal nystagmus. The ophthalmological examination revealed a reduced visual acuity in both eyes with a bilateral stage 3 papilledema at fundoscopy. Computed Tomography scan (**Figures 1A and 1B**) and MRI brain (**Figures 2A and 2B**) revealed fat in the fourth ventricle, along pre-pontine cistern, cerebellomedullary cistern, right lateral ventricle, and ambient cistern. Intensity changes in vermis were noted on CT scan and MRI (**Figures 1B and 2B**), which was possible site of dermoid cyst before rupture. Hydrocephalus was noted. We performed right ventriculo-peritoneal shunt to treat the hydrocephalus and to relieve the raised intracranial pressure. The post-operative course was uneventful. Post-operative CT (**Figure 1C**) and MRI (**Figures 2A-2C**) revealed decompressed ventricular system with shunt in situ. No significant change in lesion size was noted. He has been on follow-up for the last 12 months with no clinical, or radiological evidence of recurrence.

## Discussion

Dermoid cysts are most commonly located in the cisternal spaces, mainly in the cerebellopontine angle and parasellar cisterns. Their location in the posterior fossa is uncommon,^1^ like in our case. Posterior fossa dermoid cysts were classified by Logue and Till^3^ into 4 groups on basis of whether they were extradural or intradural, and on the degree of development of the dermal sinus, whether absent, partial, or complete: 1) extradural dermoid cyst with a complete sinus; 2) intradural dermoid cyst without a sinus; 3) an intradural dermoid cyst with an incomplete dermal sinus; 4) intradural dermoid cyst with a complete dermal sinus.^1^,^4^-^5^ Our case was type 2 according to this classification.

Clinical manifestations of posterior fossa dermoid cysts are related to mass effect-obstructive hydrocephalus (like our patient) and raised intracranial pressure.^4^ Also patients can present with headache, nausea, vomiting, papilledema, cerebellar signs such as ataxia and dysmetria, seizures, cranial nerve palsies (usually of nerves 6 and 7), bradycardia and hypertension. Aseptic meningitis secondary to the dissemination of the cyst content characterizes these tumors.^1^

Rupture of intracranial dermoid cysts is a rare phenomenon.^2^ Rupture of these cysts result in spillage of contents that may further lead to an inflammatory response, chemical meningitis, recurrent meningitis, abscess formation, extradural empyema, increased intracranial pressure, and seizure.^5^ However, the exact pathophysiological mechanism of dermoid cyst rupture is unknown. Dermoid cystic tumor rupture can occur spontaneously, or sometimes secondary to closed head trauma, or iatrogenic surgical complications. Stendel and associates^6^ hypothesized that glandular secretions, possibly increased by age, dependent hormonal changes, may lead to rapid enlargement and rupture of these cysts.^5^,^2^ Our patient had rupture of the cyst and presented with features of raised intracranial pressure.

Radiologically dermoid cysts are usually extremely hypodense on CT scan with a Hounsfield unit of -20 to -140, due to their lipid content. Calcification is frequently present, and the tumor does not enhance after the administration of contrast medium. Occasionally, they appear hyperdense mimicking a hemorrhage. On MRI, they are typically hypointense on T1-weighted image and vary from hypo- to hyperintense, and non-homogenous lesion on T2 weighted images. They typically have a high signal on fluid-attenuated inversion recovery images and are moderately restricted on diffusion-weighted images.^7^ Differential diagnosis of dermoid cyst are epidermoid cyst, arachnoid cyst, and cystic craniopharyngiomas. Demostration of fat in the dermoids and particular signal characteristics help differentiate it from the arachnoid cyst and cystic craniopharyngiomas. Epidermoids have a more variable location than dermoid cyst and are usually non-midline.^8^ Location of the lesion, low density on CT, and demonstration of the fat content on MRI support the diagnosis of dermoid in our case.

The goal of treatment in patients with dermoid cyst involve complete surgical removal of the primary tumor capsule and intra-cystic contents. Surgical management of dermoid cyst involves incising the capsule, removing cyst contents for internal debulking, and decompression, and microsurgically dissecting the capsule from adherent, or adjacent neurovascular structures. Ideally, a plane of dissection can be developed between the capsule and overlying arachnoid, but the dermoid capsules commonly have a dense adherence to the brain parenchyma and vasculature. Dermoids, in comparison to epidermoids are more adherent to arachnoid, which makes the development of a surgical plane and dissection difficult. If the tumor capsule is strongly adherent to surrounding neurovascular structures, subtotal resection should be considered, leaving the adherent portion intact to avoid vascular complications.^5^,^2^ The dissemination of lipid droplets in the subarachnoid space from spontaneous cyst rupture can be diffused and widespread, and it is not practical to remove all these droplets. Intraventricular, or subarachnoid fat does not seem to resorb and has been demonstrated to persist for years after time of rupture.^1^ Mortality and morbidity increase if chemical, or bacterial meningitis develops, or if there is associated cerebellar abscesses.^1^

In conclusion, dermoid cysts of the posterior fossa are uncommon. Patients usually present with features of intracranial hypertension, cerebellar syndrome, and sometimes with meningitis. The neuroimaging features are quite characteristic, and the presence of disseminated fat droplets in the subarachnoid apace, or ventricles on neuroimaging scans is diagnostic for a ruptured dermoid cyst.
